# Silencing the Adipocytokine NOV: A Novel Approach to Reversing Oxidative Stress-Induced Cardiometabolic Dysfunction

**DOI:** 10.3390/cells11193060

**Published:** 2022-09-29

**Authors:** Maayan Waldman, Shailendra P. Singh, Hsin-Hsueh Shen, Ragin Alex, Rita Rezzani, Gaia Favero, Edith Hochhauser, Ran Kornowski, Michael Arad, Stephen J. Peterson

**Affiliations:** 1 Cardiac Research Laboratory, Felsenstein Medical Research Center, Sackler School of Medicine, Tel Aviv University, Tel Aviv 699780, Israel; 2 Department of Pharmacology, New York Medical College, Valhalla, NY 10595, USA; 3 Department of Sports Biosciences, Central University of Rajasthan, Kishangarh 305817, India; 4 Department of Medicine, New York Medical College, Valhalla, NY 10595, USA; 5 Anatomy and Physiopathology Division, Department of Clinical and Experimental Sciences, University of Brescia, 25123 Brescia, Italy; 6 Department of Cardiology, Rabin Medical Center, Petach Tikva 49100, Israel; 7 Leviev Heart Center, Sheba Medical Center, Tel Hashomer and Sackler School of Medicine, Tel Aviv University, Tel Aviv 699780, Israel; 8 Department of Medicine, Weill Cornell Medicine, New York, NY 10021, USA; 9 Department of Medicine, New York Presbyterian Brooklyn Methodist Hospital, Brooklyn, NY 11215, USA

**Keywords:** oxidative stress, metabolic syndrome, CCN3/NOV, heme oxygenase, PGC-1, mitochondria, mitophagy, type 2 diabetes

## Abstract

Objective: NOV/CCN3 is an adipocytokine recently linked to obesity, insulin resistance, and cardiometabolic dysfunction. NOV is manufactured and secreted from adipose tissue, with blood levels highly correlated with BMI. NOV levels are increased in obesity and a myriad of inflammatory diseases. Elevated NOV levels cause oxidative stress by increasing free radicals, decreasing antioxidants, and decreasing heme oxygenase (HO-1) levels, resulting in decreased vascular function. Silencing NOV in NOV knockout mice improved insulin sensitivity. We wanted to study how suppressing NOV expression in an obese animal model affected pathways and processes related to obesity, inflammation, and cardiometabolic function. This is the first study to investigate the interaction of adipose tissue-specific NOV/CCN3 and cardiometabolic function. Methods: We constructed a lentivirus containing the adiponectin-promoter-driven shNOV to examine the effect of NOV inhibition (shNOV) in adipose tissue on the heart of mice fed a high-fat diet. Mice were randomly divided into three groups (five per group): (1) lean (normal diet), (2) high-fat diet (HFD)+ sham virus, and (3) HFD + shNOV lentivirus. Blood pressure, tissue inflammation, and oxygen consumption were measured. Metabolic and mitochondrial markers were studied in fat and heart tissues. Results: Mice fed an HFD developed adipocyte hypertrophy, fibrosis, inflammation, and decreased mitochondrial respiration. Inhibiting NOV expression in the adipose tissue of obese mice by shNOV increased mitochondrial markers for biogenesis (PGC-1α, the nuclear co-activator of HO-1) and functional integrity (FIS1) and insulin signaling (AKT). The upregulation of metabolic and mitochondrial markers was also evident in the hearts of the shNOV mice with the activation of mitophagy. Using RNA arrays, we identified a subgroup of genes that highly correlated with increased adipocyte mitochondrial autophagy in shNOV-treated mice. A heat map analysis in obese mice confirmed that the suppression of NOV overrides the genetic susceptibility of adiposity and the associated detrimental metabolic changes and correlates with the restoration of anti-inflammatory, thermogenic, and mitochondrial genes. Conclusion: Our novel findings demonstrate that inhibiting NOV expression improves adipose tissue function in a positive way in cardiometabolic function by inducing mitophagy and improving mitochondrial function by the upregulation of PGC-1α, the insulin sensitivity signaling protein. Inhibiting NOV expression increases PGC-1, a key component of cardiac bioenergetics, as well as key signaling components of metabolic change, resulting in improved glucose tolerance, improved mitochondrial function, and decreased inflammation. These metabolic changes resulted in increased oxygen consumption, decreased adipocyte size, and improved cardiac metabolism and vascular function at the structural level. The crosstalk of the adipose tissue-specific deletion of NOV/CCN3 improved cardiovascular function, representing a novel therapeutic strategy for obesity-related cardiometabolic dysfunction.

## 1. Introduction

NOV/CCN3 is one of the six members of the CCN family of proteins that regulate wound repair, angiogenesis, and cell proliferation and spreading [1]. Initially identified in chicken nephroblastomas induced by myeloblastosis associated virus type 1 (MAV-1 (N) [2], NOV/CCN3 was later found to play a role in the differentiation of cartilage [3]. Some groups showed an inverse relationship between NOV/CCN3 expression and the aggressiveness of different forms of cancer [4]. NOV/CCN3, now simply referred to as NOV, also regulates vascular smooth muscle and endothelial cell function [5].

Adipose tissue is the largest endocrine organ in the human body. The inflammation of visceral adipose tissue observed in obesity, known as adiposopathy, is key in the development of insulin resistance [6]. NOV has recently been linked to obesity, insulin resistance, and cardiometabolic dysfunction; secreted by adipose tissue, NOV is considered an adipokine, and its plasma levels are strongly correlated with BMI [7]. NOV levels are increased in obesity and a myriad of inflammatory diseases [8]. Elevated NOV levels cause oxidative stress and reduced heme oxygenase (HO-1) levels, all which result in impaired vascular function. This has been shown by our group both in obesity and sleep apnea [9,10,11]. The chronic inflammatory state of obesity is associated with the overproduction of oxidative free radicals and the generation of superoxides [12]. This state of oxidative stress alters cell signaling, cellular processes, and transport mechanisms, all of which contribute to the development of insulin resistance [13,14]. Adipose tissue inflammation leads to lipotoxicity, which, in turn, interferes with metabolic pathways in adipose tissue and also in distant organs such as the liver, heart, pancreas, and skeletal muscle. Altogether, insulin resistance and visceral adiposity are responsible for cardiometabolic dysfunction in obesity [15].

Obesity and metabolic syndrome often lead to heart failure (HF) [16]. In humans, obesity leads to the accumulation of epicardial adipose tissue and adipose tissue in the liver, kidney, and other organs [16,17,18]. Peripheral and visceral adiposity are a significant source of reactive oxygen species (ROS) and inflammation, which exacerbate and perpetuate the cardiac, liver, and metabolic disfunctions associated with obesity and metabolic syndrome. [19,20,21,22,23].

The induction of the antioxidant enzyme Heme oxygenase-1 (HO-1) or the upregulation of its nuclear co-activator, PGC1 α, confers advantageous effects on metabolic syndrome [24,25,26]. HO-1 acts through heme degradation products with antioxidant properties that increase mitochondrial fusion [26]. It also improves adipocyte and vascular functions by increasing adiponectin expression and reducing inflammation [27]. The induction of HO-1 in adipose tissue reduces body weight and NOV expression and increases PGC-1α-mediated thermogenesis, with resultant increased energy uptake and mitochondrial fatty acid (FA) oxidation [26,28]. In adipose tissue, PGC-1α promotes mesenchymal stem cell differentiation into brown/beige fat with a distinct phenotype, which is rich in mitochondria, increasing energy metabolism and preventing the development of metabolic syndrome and type 2 diabetes mellitus (T2DM) [29,30,31].

We have previously shown that weight reduction reduces NOV levels and improves mitochondrial function, with an increase in brown adipose tissue and improved mitochondrial signaling and distant organ function [11]. Based on these findings, we hypothesize that reducing NOV levels in an animal model of obesity will improve cardiometabolic function and signaling/cellular processes linked to obesity. In the present studies, we selectively silenced NOV in adipose tissue by inoculating mice with Adipo -shNOV lentivirus, producing NOV silencing in adipocytes to study the effect on inflammatory markers and its impact on cardiometabolic function.

## 2. Materials and Methods

### 2.1. Experimental Animals and the Generation of Lentiviral Vector for NOV Silencing in Mice

Animal experiments were performed according to procedures approved by the Institutional Animal Care and Use Committee (IACUC) of the New York Medical College. (Protocol #22-2-0415H, final approval 1 July 2020).

Sixteen week-old male mice on a C57BL/6J background (Jackson Labs, Bar Harbor, ME, USA) were randomly divided into three groups (five mice per group) as follows: (1) lean + normal diet, (2) HFD, HFD: mice fed an HFD for 28 weeks + sham (inactive, placebo) virus for weeks 20–28, and (3) HFD for 28 weeks + shNOV lentivirus for weeks 20–28. At 20 weeks of an HFD, the mice in group ‘3′ were administered a bolus injection of 80–100 μL Adipo-shNOV lentivirus (40–70 × 109 TU/mL in saline); untreated HFD mice (group ‘2′) were similarly injected with Lnv-adipo-GFP control vector; and lean mice (group ‘1′) received mock virus (placebo). The mice in groups 2 and 3 were kept on an HFD for an additional 8 weeks from weeks 20 to 28. The high-fat diet (HFD) consisted of: 58% fat (from lard), 25.6% carbohydrate, and 16.4% protein (total calories: 23.4 KJ/g) (Bio-SERV, Frenchtown, NJ, USA), and the lean mice were fed, ad libitum, a normal chow diet containing 11% fat and 62% carbohydrates, as in [26]. Lentiviral vectors under an adiponectin-specific promoter, expressing either shNOV or control vector, were constructed using the LentiMax^TM^ system (Lentigen, Baltimore, MA, USA and Vector Builder, Shenandoah, TX, USA.

### 2.2. Physiological Assessment

Mice were allowed to acclimatize in the oxygen consumption chambers for a three-week period (2 h, three times every week). Each mouse was placed individually in the Oxylet gas analyzer and airflow unit (Oxylet, Panlab-Bioseb, Vitrolles, France). To determine oxygen consumption; VO_2_, VCO_2,_ and respiratory quotient (RQ) were calculated as VCO_2_/VO_2_, as previously described [26]. Fasting blood glucose was measured using a standard glucometer, and blood pressure was measured using the tail-cuff method, as described [26,32,33].

### 2.3. Assessment of Vasorelaxation in Renal Interlobar Artery Rings (Myograph)

To assess vascular function, we measured vasoconstriction and vasorelaxation in the renal interlobar arteries. Arteries were cut into ring segments (2 mm in length) and mounted on 40 µm stainless steel wires in chambers of a myograph (J.P. Trading, Aarhus, Denmark), for the measurement of isometric tension, in a bath (37 °C) containing Krebs buffer supplemented with indomethacin (1 μmol/L) and gassed with 95% O_2_-5% CO_2_. After a 30 min equilibration, the rings were set to an internal circumference equivalent to 90% of the relaxed circumference under a transmural pressure of 100 mmHg and were allowed to stabilize for 20–30 min. The rings were then depolarized with KCl (60 mM) to evaluate the maximal contraction. After washing, the vessels were contracted with increasing concentrations (10^–6^ M) of phenylephrine followed by vasorelaxation responses to cumulative increments (10^−8^ to 10^−4^ mol/L) of Acetylcholine [26,34].

### 2.4. Generation of NOV-Overexpressing and NOV Deficient Adipocyte Cells Using Lentiviral Vectors

3T3-L1 mouse pre-adipocytes were purchased from the ATCC (Manassas, VA, USA). After thawing, 3T3-L1 cells were cultured as previously described [26,35]. For studies aimed at achieving adipocyte cell-specific overexpression or knockdown of NOV, 1 × 10^6^ cells were seeded in six-well plates. Adipocyte (3T3-L1) cells were transfected for 3 h with 1 × 10^6^ transducing units (TF) of overexpressing NOV (ORF-NOV) or NOV knockdown (shNOV) lentivirus (Vector builder, Shenandoah, TX, USA) to establish a stably transduced cell line. Cells were also treated with the transduction medium without lentiviral particles, which served as the un-transduced control. After 48 h of incubation, the antibiotic selection medium (α-MEM growth medium with 10 µg/mL puromycin) was used to select for transduced cells. Cells were then cultured and maintained as described above [26,32,33].

### 2.5. Measurement of Oxygen Consumption Rate in Adipocyte Cells

The oxygen consumption rate (OCR) was measured as oxygen consumption per minute (pmols/min). Additionally, the extracellular acidification rate (ECAR) was recorded and is a measure of glycolysis; the units are (mpH/min). The extracellular flux analyzer XFp (Seahorse Bioscience, Houston, TX, USA) was used to measure OCR in the cells, which were overexpressed, and the knockdown for PGC-1α. Adipocytes derived from 3T3-L1 cells were plated at 4 × 10^5^ cells/well into the Seahorse 8-well microplate. Oligomycin, FCCP, rotenone, and antimycin were freshly prepared in XF assay media. Antimycin A is an inhibitor of ATP synthase, so OCR reduction after antimycin A treatment represents ATP turnover under the specified conditions. FCCP is an uncoupling agent of electron transport and can generate a proton efflux to induce the maximum respiration termed as respiratory capacity or uncoupled respiration [26].

### 2.6. Western Blot Analysis

Western blot analysis was performed as previously described [36]. At the end of the experimental diet regimens, mice were euthanized, and desired tissues were snap-frozen in liquid nitrogen and stored at −80°C. Frozen mouse adipose, heart, and liver tissue were homogenized in lysis buffer. Proteins were added to acrylamide gels and transferred to nitrocellulose membranes via the Trans Blot Turbo transfer machine (Bio-Rad, Hercules, CA, USA). Immunoblotting for PGC-1α, MFN1, Fis1, UCP1, TWIST1, FAK, NOV, and others was performed using specific antibodies (Cell Signaling Technology, Danvers, MA, USA). Membranes were incubated overnight with the primary antibody, followed by LI-COR-specific secondary antibodies and detection with a LI-COR Odyssey infrared imaging system (LI-COR, Lincoln, NE, USA) [37,38,39].

### 2.7. Immunofluorescence

The adipose tissue-collected samples were fixed in 4% buffered paraformaldehyde for 24 h before being conventionally dehydrated and embedded in paraffin wax. Serial sections (7 µm thick) of each sample were cut with a microtome and submitted to PGC-1α immunofluorescence evaluations. In detail, alternate adipose tissue paraffin sections were deparaffinized, rehydrated, and then incubated with polyclonal anti-rabbit PGC-1α antibody (diluted 1:400; Abcam, Cambridge, UK). After washing, the sections were labeled with specific Alexa Fluor-conjugated secondary antibodies (diluted 1:200; Invitrogen–Thermo Fisher Scientific, IL, USA), and then the sections were counterstained with 4′ -6-diamidino-2- phenylindole (DAPI), mounted, and observed with fluorescent microscopy (i50 Eclipse, Nikon, Hamburg, Germany) at a final magnification of 400×. Sections without a primary antibody and in the presence of isotype-matched IgG served as negative immunofluorescence controls. Twenty random fields from a total of five non-consecutive sections per animal were analyzed, and the nuclear PGC-1α immunostaining was calculated using an image analyzer (Image Pro Premier 9.1, Media Cybernetics, Rockville, MD, USA) and expressed in AU. Two blinded investigators performed the analysis, and their evaluations were assumed correct if the values were not significantly different. If there was disagreement concerning the interpretation, the case was reconsidered in order to reach a unanimous agreement.

### 2.8. RT-PCR and RNA Arrays

Total RNA was obtained from frozen tissues, as previously described [36], using RNeasy Lipid Tissue (Qiagen, Hilden, Germany), according to the manufacturer’s instructions. Gene expression analysis levels were determined using the relative expression method with the threshold crossing point (Ct-value), as described [26,30,37].

PCR arrays for the RT^2^ Profiler™ PCR Array Mouse Adipogenesis (Qiagen, product no. 330,231 and Cat. No. PAMM-049Z) were performed following the manufacturers’ protocol. Gene expression levels were calculated using the ΔΔCt method after normalization to the housekeeping gene expression and determination of the fold change. Each GeneQuery™ plate contains eight controls, five target housekeeping genes (β-actin, GAPDH, LDHA, NONO, and PPIH), and genes encoding for pre-adipocyte cell markers, proliferation, differentiation and adipogenesis, lipid metabolism, and obesity. Gene expression is presented as the log10 of mean values (*n* = 3 in each group), as previously described [26,40,41] (https://CRAN.R-project.org/package=gplots, accessed on 4 March 2022).

### 2.9. Statistics

In the animal studies, the results were normalized to the control group (lean mice) and presented as the means ± standard errors of the means (SEM), with individual values indicated. For comparison between two groups, an unpaired *t*-test was performed. For the comparison of more than two groups, statistical analysis was performed with one-way analysis of variance (ANOVA) followed by the Newman–Keuls post hoc multiple-comparison method. *p* values of 0.05 or smaller were considered statistically significant. Significance was depicted by stars: * *p* < 0.05; ** *p* < 0.01; *** *p* < 0.001; and **** *p* < 0.0001.

## 3. Results

### 3.1. Overexpression of NOV Promotes Adipocyte Differentiation

**Figure 1:**
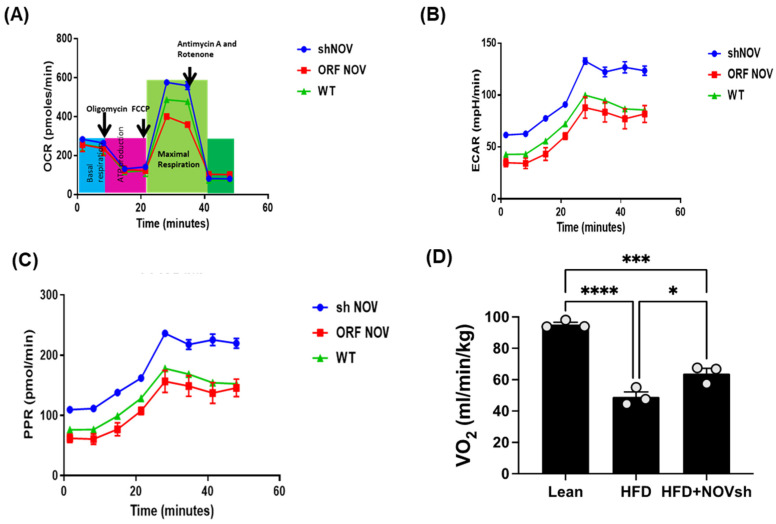
Mitochondrial function and inflammation in adipocytes with the overexpression of NOV (ORF NOV) or NOV knockdown (shNOV) in mice on an HFD. Oxygen consumption rate (OCR) (**A**), Extra Cellular Acidification Rate (ECAR) (**B**), and proton production rate (PPR) (**C**) were measured in differentiated adipocytes with the silencing of NOV (shNOV) or the overexpression of NOV (ORF NOV). Oxygen consumption (VO_2_) in lean, HFD, and HFD + shNOV mice. (**D**). Results are means ± SE; * *p* < 0.05, *** *p* < 0.001, **** *p* < 0.0001 by one-way ANOVA followed by multiple comparisons.

**Figure 2:**
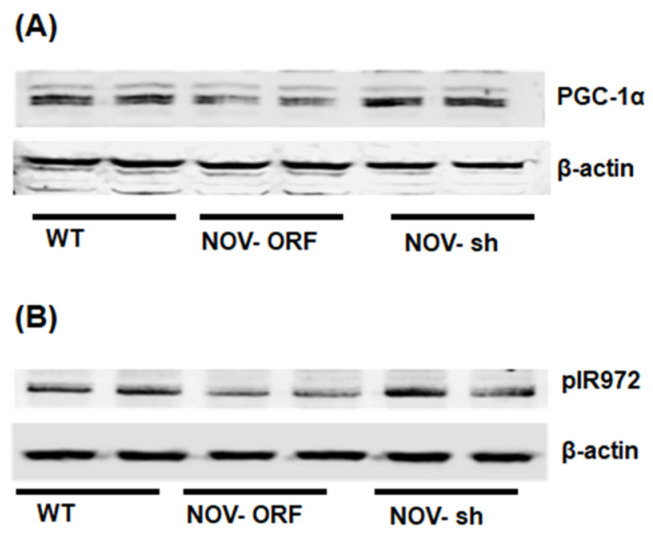
Mitochondrial function, inflammation, and insulin signaling in adipocytes with the overexpression of NOV (ORF NOV) or NOV knockdown (shNOV) in mice on an HFD. Western blot analysis of PGC-1α (**A**), phosphorylated insulin receptor (pIR972) (**B**). Results are the average of two experiments run in duplicates.

**Figure 3:**
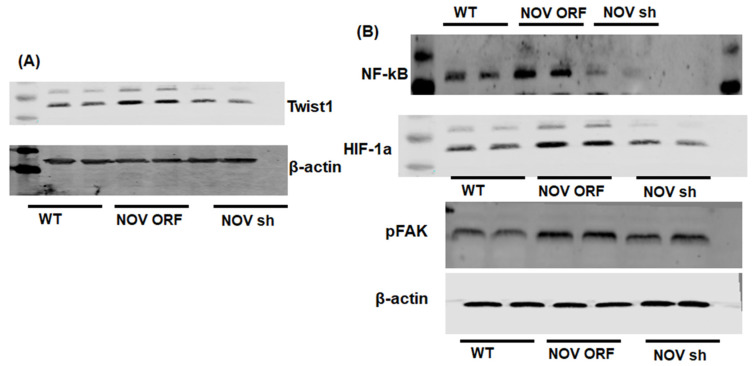
Mitochondrial function and inflammation in adipocytes with the overexpression of NOV (ORF NOV) or NOV knockdown (shNOV) in mice on an HFD. Western blot analysis of Twist Family BHLH Transcription Factor 1 (TWIST1) (**A**), nuclear factor kappa-light-chain-enhancer of activated B cells (NF-ƙB) (**B**), hypoxia-inducible factor 1-alpha (HIF 1α) (**B**), and phosphorylated focal adhesion kinase (pFAK) (**B**). Results are the average of two experiments run in duplicate.

To verify the efficacy of NOV transfection and knockdown, we used adipocytes overexpressing NOV (NOV-ORF), or NOV knockdown (shNOV), cultured in adipogenic differentiation media. These in vitro experiments were performed twice in duplicate. Figure 1, Figure 2 and Figure 3 are the in vitro studies showing that silencing NOV could be achieved. Therefore, the first three figures were the average of two experiments, run in duplicate, to illustrate the innovation in viral construction. The experiment showed that NOV could be silenced. The second aim was to demonstrate the success of NOV gene suppression, as in in vivo studies. Our data showed, for the first time, statistically significant results, which are presented in western blot and dot plot fashion. As shown in Figure 1, the maximal respiration rate was reduced in adipocytes overexpressing NOV (ORF NOV) compared to control (WT) cells (Figure 1A). On the contrary, suppressing NOV expression (shNOV) elevated the maximal respiration rate above that of the control cells (Figure 1A). Both the extracellular acidification rate (ECAR) and proton production rate (PPR) were also reduced in the NOV overexpressing cells and increased in the shNOV cells (Figure 1B,C). The average VO_2_ values for the groups of animals including shNOV mice are summarized in Figure 1D. The overexpression of NOV decreased the levels of PGC-1α (*p* < 0.05), as well as the phosphorylation of the insulin receptor (pIR972) (Figure 2B), whereas the suppression of NOV increased the expression (*p* = NS) of these proteins (Figure 2B). The pro-inflammatory proteins NF-ƙB, TWIST1, FAK, and HIF-1α were also upregulated in the ORF-NOV adipocytes (*p* = NS) but downregulated (*p* = NS) in the shNOV cells (Figure 3A,B). Altogether, these results suggest that NOV increases inflammation and impairs mitochondrial function—key processes in adipogenesis.

### 3.2. Adipocyte-Specific In Vivo Silencing of NOV

**Figure 4:**
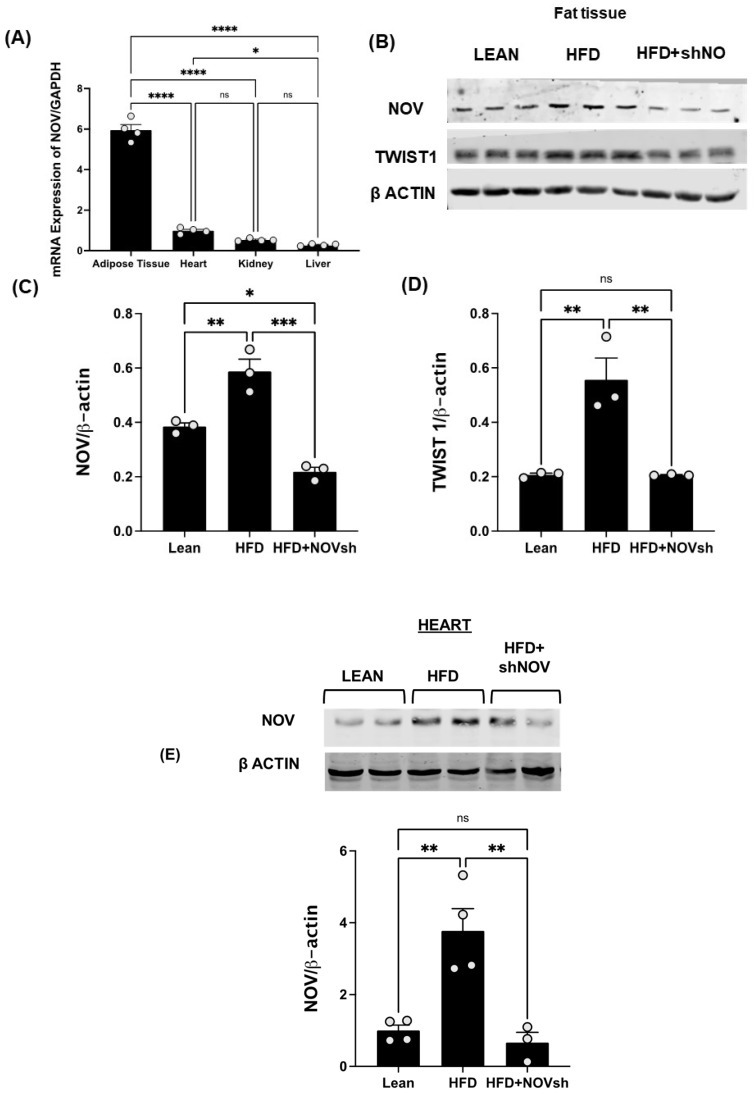
In vivo activation of NOV in the adipose tissue of mice fed an HFD: effects of NOV knockdown (shNOV) treatment. mRNA levels of NOV in the adipose tissue, heart, kidney, and liver (**A**), * *p* < 0.05 versus adipose tissue in mice on an HFD or Adipo- shNOV transgenic mice on an HFD N = 4, * *p* < 0.05 vs. HFD mice. Representative western blots and quantification of NOV in adipose tissue and TWIST1 in adipose tissue (**B**–**D**) of lean, high-fat, and shNOV mice. * *p* < 0.05 versus lean. Representative western blot and quantification of NOV protein levels in the hearts (**E**) of lean, HFD, and HFD+shNOV mice. Results are means ± SE; * *p* < 0.05, ** *p* < 0.01, *** *p* < 0.001, **** *p* < 0.0001, ns = not significant, *n* = 3.

To test if the upregulation of NOV expression is prevented by the adipocyte-specific silencing of NOV in obese mice, we first compared the expression levels of NOV in fat and other mouse tissues. The NOV levels were markedly higher in adipose tissue compared to its expression in the heart (6-fold, *p* < 0.001), liver (20-fold, *p* < 0.001), and kidney (11-fold, *p* < 0.001) (Figure 4A), suggesting that the function of NOV is more important in adipose tissue compared to other organ systems. We then silenced NOV expression in the fat tissue of mice fed an HFD. Using RT-qPCR, we confirmed that the adipo-shNOV transgenic mice have a lower expression of NOV—specifically, in adipose tissue (Figure 4B) and in the heart (Figure 4E).

The HFD regimen elevated the NOV gene and protein expression levels in adipose tissue compared to those in lean animals (Figure 4B,C, *p* < 0.05). Importantly, NOV protein levels were reduced in shNOV-treated mice (Figure 4B,C *p* < 0.05). In line with these findings, the NOV downstream pro-inflammatory protein TWIST1 was increased (*p* < 0.05) in the HFD-fed mice and markedly attenuated in the shNOV-treated animals (Figure 4B,D *p* < 0.05).

### 3.3. NOV Silencing Promotes the Translocation of PGC-1α to the Nucleus

**Figure 5:**
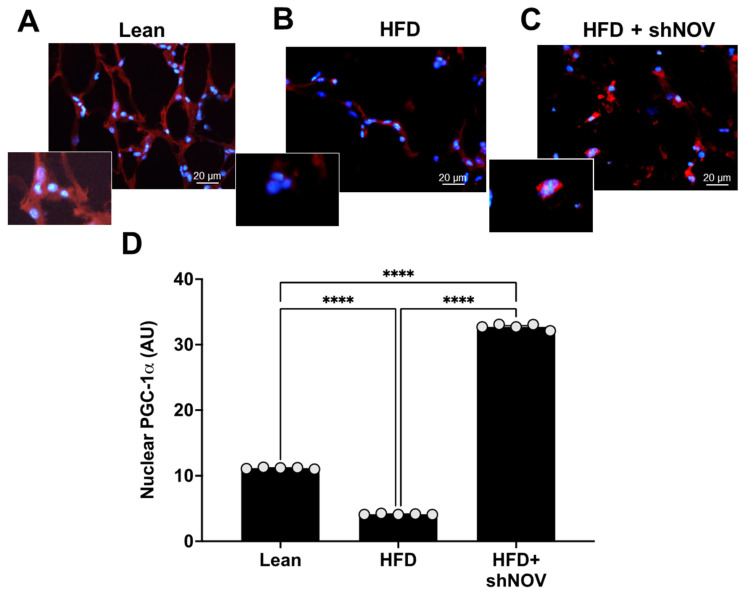
Immunofluorescence of PGC-1α expression (red staining) in adipose tissue. Immunofluorescence photomicrographs of PGC-1α expression (red staining) in the adipose tissue of lean (**A**), HFD (**B**), and HF + shNOV (NOV knockdown) (**C**) mice. Bar 20 μm. The graph summarizes the nuclear expression of PGC-1α in the adipose tissue (**D**) of lean, high-fat, and shNOV-treated mice. Results are means ± SE; **** *p* < 0.0001.

For PGC-1α to act as a transcriptional co-activator, it must be present in the nucleus. Immunofluorescence was performed to show the co-localization and expression of PGC-1α in the nucleus of adipocyte cells in the lean, HFD, and shNOV mice. In the adipose tissue from HFD mice, the presence of PGC-1α was reduced (*p* < 0.0001) compared to lean mice. Importantly, shNOV mice fed an HFD showed a significant increase (*p* < 0.0001) in PGC-1α nuclear localization when compared to the HFD group (Figure 5A–D).

### 3.4. Suppression of NOV Expression Improves Oxygen Consumption and Attenuates Vascular Stiffness

**Figure 6:**
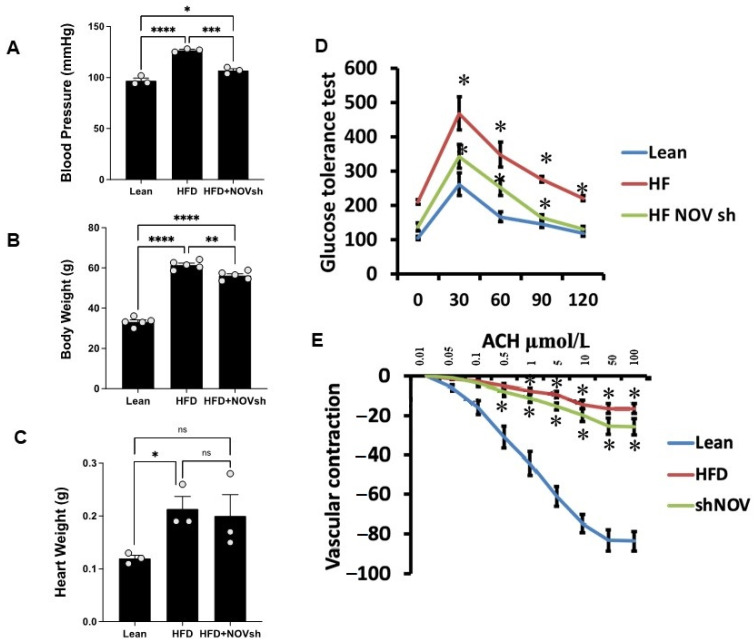
Blood pressure, body weight, heart weight, and glucose tolerance are all improved in shNOV-treated mice (NOV knockdown) on an HFD followed by the activation of PGC-1α, with an improvement in metabolic syndrome. Blood pressure (**A**), body weight (**B**), heart weight (**C**), glucose tolerance (**D**), and vascular contraction (**E**), of lean, high-fat, and shNOV-treated mice. Results are means ± SE; * *p* < 0.05, ** *p* < 0.01, *** *p* < 0.001, **** *p* < 0.0001, ns = not significant.

Mice on an HFD showed increased blood pressure (*p* < 0.0001) and body weight and impaired glucose tolerance compared to lean mice (Figure 6A–D). In HFD mice treated with shNOV (NOV knockdown), the elevations in blood pressure and body weight were significantly attenuated (*p* < 0.05) (Figure 6A,B). The inhibition of NOV was not sufficient to induce HO-1 in order to reduce heart weight (Figure 6C). When HO-1 was added with NOV, it was sufficient to reduce heart weight (*p* < 0.05).

Glucose tolerance was also markedly improved (Figure 6D). The HFD regimen decreased oxygen consumption (VO_2_) compared to lean mice (*p* < 0.05) but was higher in the shNOV-treated animals (*p* < 0.05, Figure 1D). When the vascular function was examined, we found a reduced contractility in response to acetylcholine in vessels from HFD-fed mice, which was improved in the shNOV-treated animals (Figure 6E). Since obesity promotes cardiac hypertrophy, it was not surprising that mice fed an HFD exhibited increased heart weight (*p* < 0.05). Again, this was attenuated in shNOV-treated mice.

### 3.5. Silencing NOV Increases the Expression of PGC-1α, the Nuclear Co-Activator of HO-1

**Figure 7:**
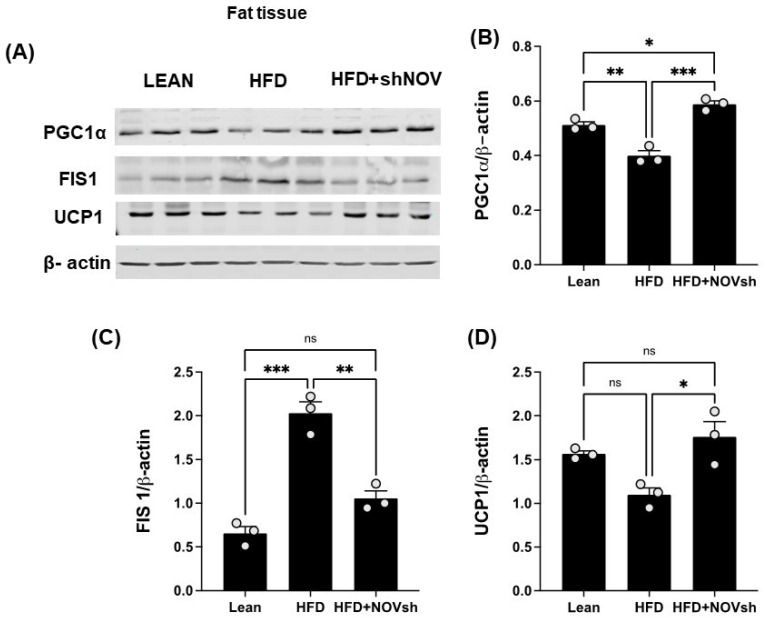
In vivo silencing of NOV with shNOV treatment (NOV knockdown) improves mitochondrial protein levels and metabolic markers in the adipose tissue. Representative western blot of (**A**): PGC1α (peroxisome proliferator-activated receptor-gamma coactivator), FIS1 (mitochondrial fission 1 protein), and UCP1 (uncoupling protein 1), and corresponding quantifications (**B**–**D**) in the adipose tissue of lean, high-fat, and shNOV mice. Results are means ± SE; * *p* < 0.05, ** *p* < 0.01, *** *p* < 0.001, ns = not significant.

Since our data displayed an improvement in oxygen consumption in the shNOV-treated mice, we next measured the expression levels of mitochondrial proteins in adipose tissue, which, as we have previously shown, are involved in adipocyte function [41]. As shown in (Figure 7A,B), PGC-1α levels, which were downregulated in the HFD-fed mice, were upregulated in the shNOV-treated animals. FIS1, a protein that regulates mitochondrial fission, was elevated (*p* < 0.0001) in the HFD-fed mice, but its levels were restored (*p* < 0.01) to those in lean mice after shNOV treatment (Figure 7A,C). A similar pattern of change was observed for UCP1 (Figure 7A,D). Altogether, these results demonstrate that the dysregulation of mitochondrial proteins in adipose tissue, which contributes to adipocyte dysfunction, can be prevented by the inhibition of NOV.

**Figure 8:**
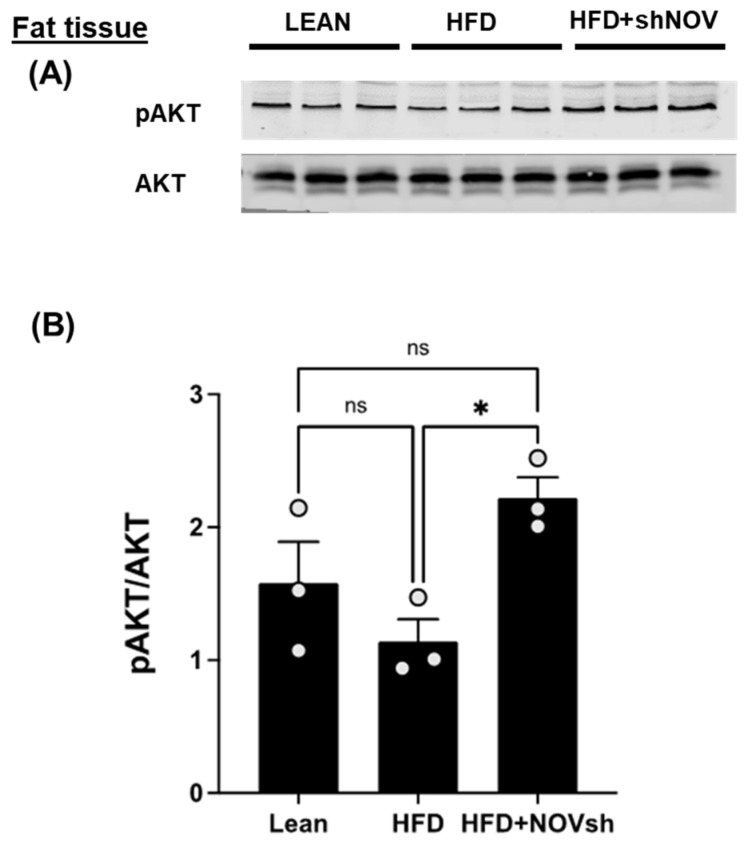
In vivo silencing of NOV with shNOV treatment (NOV knockdown) improves metabolic markers in adipose tissue. Representative western blot of pAKT and AKT (protein kinase B) (**A**), and corresponding quantification of the pAKT/AKT ratio (**B**) in the adipose tissue of lean, high-fat, and shNOV mice. Results are means ± SE; * *p* < 0.05, ns = not significant.

The cellular energy status regulates the activation of AKT, a key component of insulin signaling. In the adipose tissue of HFD-fed mice, the activation of AKT, as assessed indirectly from the extent of their phosphorylation, was reduced by a high-fat diet, but not significantly. Silencing NOV significantly increased AKT compared to that in lean mice (*p* < 0.05), but it was restored in mice on an HFD treated with shNOV (NOV knockdown) (Figure 8A,B).

### 3.6. In vivo Suppression of NOV Expression Improves Mitochondrial Markers in the Heart with the Concomitant Upregulation of Mitophagy

**Figure 9:**
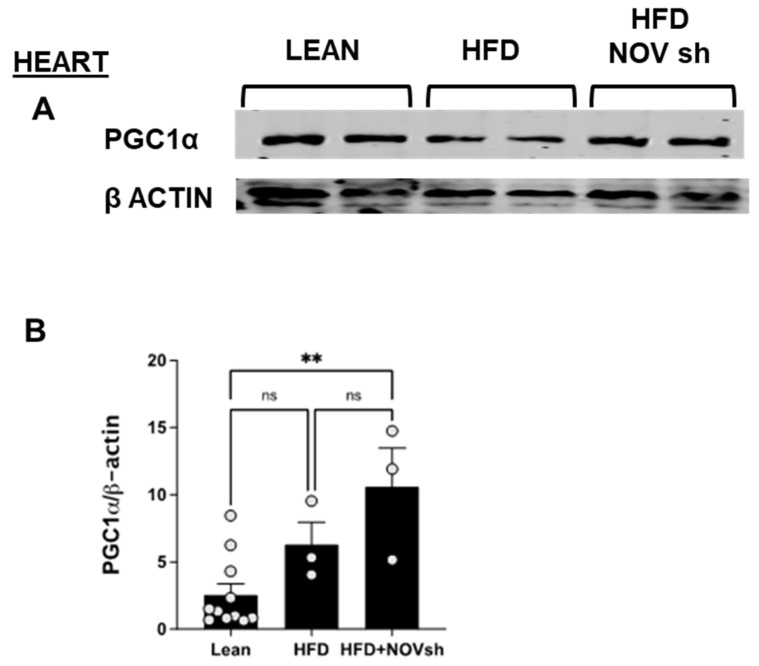
Improved expression of mitochondrial proteins in the heart of shNOV-treated mice (NOV knockdown). Representative western blots of PGC-1α (**A**,**B**), and corresponding quantifications (**B**) in the heart of lean, high-fat, and shNOV. Results are means ± SE; ** *p* < 0.01, ns = not significant.

To examine if the improvement in adipose tissue function following NOV inhibition is also revealed in the heart, we measured mitochondrial and antioxidant proteins in cardiac tissue. PGC-1α levels were reduced (*p* < 0.05) in HFD-fed mice compared to lean animals’ PGC-1α in cardiac tissue (Figure 9A). Notably, diminishing NOV expression restored the expression of this protein to the levels shown by lean animals (*p* < 0.05, Figure 9A,B).

### 3.7. RNA Array Analyses of Gene Expression in Adipose Tissue

**Figure 10:**
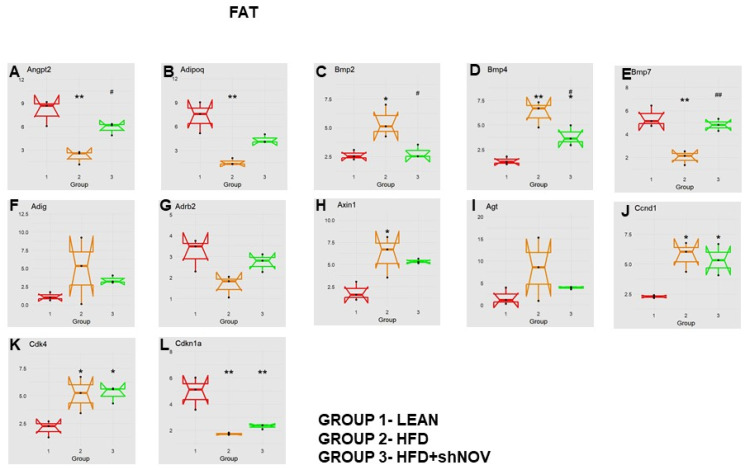
Changes in gene expression in the adipose tissue of shNOV-treated mice (NOV knockdown). RNA-array-analyses changes in the correlation coefficients of gene expression in lean, HFD, and Lnv-adipo-shNOV (HFD) groups. Shown is the mRNA expression (PCR array) of Angiopoietin 2 (Angpt2) (**A**), adiponectin (Adipoq) (**B**), Bone Morphogenetic Protein 2 (BMP2) (**C**), Bone Morphogenetic Protein 4 (BMP4) (**D**), Bone Morphogenetic Protein 7 (BMP7) (**E**), Adipogenin (Adig) (**F**), beta-2-adrenergic receptor (Adrb2) (**G**), Axin (**H**), Angiotensinogen (Agt) (**I**), Cyclin D1 (Ccnd1) (**J**), Cyclin-Dependent Kinase 4 (Cdk4) (**K**), and Cyclin-Dependent Kinase Inhibitor 1A (Cdkn1a) (**L**) in the adipose tissue of lean, high-fat diet (HFD), and shNOV-treated mice. Data are expressed as means ± SE; (*n* = 5); * *p* < 0.05, ** *p* < 0.005 versus lean; # *p* < 0.05, ## *p* < 0.005 versus HFD.

**Figure 11:**
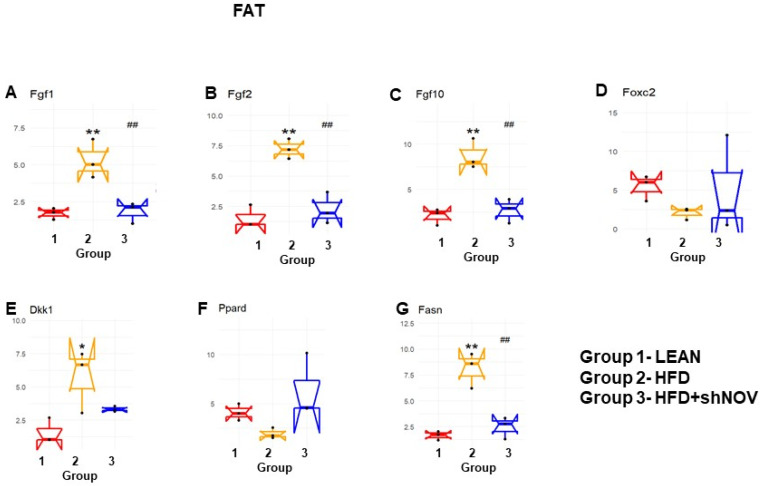
Changes in gene expression in the adipose tissue of shNOV-treated mice (NOV knockdown). RNA-array-analyses changes in the correlation coefficients of gene expression in lean, HFD, and Lnv-adipo-shNOV (HFD) groups. Shown are the mRNA expression (PCR array) Fibroblast growth factors (FGF) 1 (**A**), FGF 2 (**B**), FGF 10 (**C**), Forkhead transcription factor C (FOXC2) (**D**), Dickkopf-related protein 1 (DKK1) (**E**), Peroxisome Proliferator-Activated Receptor Delta (PPARD) (**F**), and fatty acid synthase (FASN) (**G**) in the adipose tissue of lean, high-fat diet (HFD), and HFD+shNOV mice. Data are expressed as means ± SE; (*n* = 5); * *p* < 0.05, ** *p* < 0.005 versus lean; ## *p* < 0.005 versus HFD.

Our study investigated the role of NOV during adipogenesis. We analyzed the expressions of 88 genes that are expressed only in adipocytes before and during adipogenesis, as detailed in [33]. As shown, the mRNA levels of Angiopoietin 2 (Angpt2) (Figure 10A), adiponectin (Adipoq) (Figure 10B), Bone Morphogenetic Protein 7 (BMP 7) (Figure 10E), beta-2-adrenergic receptor (Adrb2) (Figure 10G), and Cyclin-Dependent Kinase Inhibitor 1A (Cdkn1a) (Figure 10L) were downregulated in the adipose tissue of HFD-fed mice compared to lean mice (*p* < 0.05) but upregulated (*p* < 0.05) in shNOV-treated animals. BMP2/4 (Figure 10C,D), Adipogenin (Adig) (Figure 10F), Axin (Figure 10H), Angiotensinogen (Agt) (Figure 10I), Cyclin D1 (Ccnd1) (Figure 10J), and Cyclin-Dependent Kinase 4 (Cdk4) (Figure 10K) were all upregulated in the adipose tissue derived from HFD-fed mice (*p* < 0.05) but downregulated in shNOV-treated animals (*p* < 0.05). In addition, we measured the expression of several genes involved in cellular development and metabolism (Figure 11A–G). The mRNA levels of fibroblast growth factors (FGF) 1, 2, and 10 were increased in the HFD-fed mice (*p* < 0.005), whereas this upregulated expression was attenuated in HFD-fed mice treated with Lenti-adipo-shNOV (*p* < 0.005) (Figure 11A–C). The Forkhead transcription factor C (FOXC2) was downregulated in HFD-fed mice but increased in the shNOV-treated animals (Figure 11D). Dickkopf-related protein 1 (DKK1) expression was increased in the HFD group (*p* < 0.05). Adipo-shNOV treatments attenuated this upregulation (Figure 11E). Peroxisome Proliferator-Activated Receptor Delta (PPARD) and fatty acid synthase (FASN) are involved in fatty acid metabolism and synthesis. PPARD was downregulated in the HFD mice, and this effect was attenuated by the silencing of NOV (Figure 11F). FASN levels were elevated in the HFD group (*p* < 0.005), and Lenti-adipo-shNOV treatments attenuated this upregulation (Figure 11G, *p* < 0.005).

## 4. Discussion

The present study provides experimental evidence showing that the selective inhibition of the adipokine NOV in the fat tissue of obese mice ameliorates metabolic syndrome, oxidative stress, and inflammation. This is evident from the reduced weight loss, heart weight, and blood pressure and the improved insulin sensitivity in those animals. These changes were accompanied by increased oxygen consumption and vascular responsiveness and reduced adipocyte hypertrophy. At the molecular level, the inhibition of NOV led to AKT activation, mitochondrial fission, increased antioxidant defenses, and the activation of mitophagy.

NOV plays a pivotal role in the regulation of inflammation, oxidative stress, and fibrosis [7]. The upregulation of NOV has been linked to the development of obesity and insulin resistance [42,43]. In humans, circulating levels of NOV show a positive correlation with obesity and metabolic syndrome [8,9]. Moreover, NOV is highly expressed in the epicardial fat of obese patients [44]. Several studies have shown that NOV expression is reduced in conditions where HO-1 and PGC-1α are upregulated, which positively correlates with improved mitochondrial function and reduced inflammation [11,28,33,36]. In particular, the repression of NOV by inducing HO-1/PGC-1α or by overexpressing HO-1 markedly attenuates body weight, improves cardiac function, and reduces liver steatosis [40]. The observed reduction in adipocyte size and fibrosis and the upregulation of PGC-1α, which resulted in weight loss and improved oxygen consumption in shNOV-treated animals, suggest the conversion of white adipose tissue to a healthier brown fat [40]. Importantly, a large clinical trial showed that individuals with higher brown adipose tissue have a lower prevalence of diabetes, dyslipidemia, and cardiovascular disease [45].

It is well known that insulin resistance and impaired vascular relaxation contribute to cardiac hypertrophy, eventually leading to heart failure [46]. Mitochondrial dysfunction causes oxidative stress, which leads to the development of cardiomyopathy, as demonstrated in PGC-1α KO mice with reduced ATP generation [47]. Furthermore, knocking down PGC-1α aggravates the cardiac hypertrophy induced by aorta constriction [48]. Additionally, the muscle from diabetic patients exhibits reduced levels of PGC-1α and mitochondrial genes [49]. PGC-1α protects the heart by improving adipocyte function and vascular tone; indeed, we have previously demonstrated that a reduction in PGC-1α levels in the visceral fat impairs cardiac function [28,44,50]. Our results suggest an inhibitory interaction between the levels of NOV and PGC-1α: inhibiting NOV upregulated PGC-1α levels in vitro and in obese mice (Figure 2A,B, Figure 7A,B and Figure 9A,B), improving oxygen consumption, upregulating metabolic and mitochondrial markers in the heart, and attenuating heart size. All these observations strongly suggest that the inhibition of NOV can protect the heart against metabolic perturbation and the development of hypertrophy.

To gather insight into the molecular basis of the impact of NOV silencing on high-fat diet-induced obesity, we studied the differential gene expression in adipose tissue in mice fed a high-fat diet. This information is of high importance in identifying targets for human weight loss. The examination of transcriptomic changes in adipocytes in vivo when NOV expression was reduced showed the upregulation of several genes involved in energy production, ATP synthesis, mitochondrial function, and regulation of autophagy. Of note, this analysis revealed an upregulation of angiopoietin 2, which was demonstrated to promote a healthier expansion of adipose tissue, with reduced inflammation and improved glucose tolerance and lipid clearance [51].

AMPK activation leads to the phosphorylation of AKT and the inhibition of mTOR through tuberous sclerosis 1/2 (TSC1/2) complex, activating autophagy. The impairment of both lipophagy and autophagy is associated with the development of hepatic steatosis and cardiac hypertrophy [52], whereas the induction of autophagy leads to a reduction in steatosis [53]. Noteworthy ezetimibe and statins lead to an increase in autophagic flux and a consequent reduction in hepatic inflammation and fibrosis [54]. Thus, the modulation of autophagy in a manner that is beneficial in the setting of obesity and metabolic syndrome may offer a therapeutic approach for metabolic syndrome-associated cardiovascular complications.

Mitophagy selectively degrades damaged mitochondria to preserve mitochondrial dynamics and function [55]. Based on the present findings, we suggest that mitophagy might be the culprit behind the improved mitochondrial function in shNOV-treated mice. In the shNOV-treated animals, AKT, which promotes autophagy, was activated. Thus, it is possible that the upregulation of mitophagy following the inhibition of NOV leads to improved oxygen consumption by promoting fatty acid oxidation, attenuating adiposity, and improving insulin sensitivity and vascular reactivity. More studies are needed to confirm this. These changes are expected to have a major impact on metabolic syndrome and the risk of cardiovascular disease [56]. Our results strongly support the notion that selectively targeting the adipose tissue phenotype through NOV may represent an alternative strategy to treat obese patients with cardiovascular co-morbidities. In addition, we found that genes related to lipid metabolism were upregulated, while genes related to the redox state were downregulated. It is thus plausible that a similar pattern of gene expression exists in humans consuming high-fat diets. RNA arrays (88 genes) in the visceral fat from HFD-fed Lnv-adipo-shNOV mice revealed a gene expression pattern that provides insight into the potential mechanisms by which NOV protein silencing prevents HFD-driven metabolic dysfunction. The data in Figure 10 and Figure 11 demonstrates that silencing NOV reverses the HFD-induced downregulation of Angpt2, Adiponectin, PPARD, Foxc2, and mitochondrial proteins, improving cardiometabolic function through improved cardio bioenergetics. This new group of genes and biomarkers thus emerges as a group of promising targets for the treatment of obesity-related metabolic diseases and cardiomyopathies.

## 5. Conclusions

**Figure 12:**
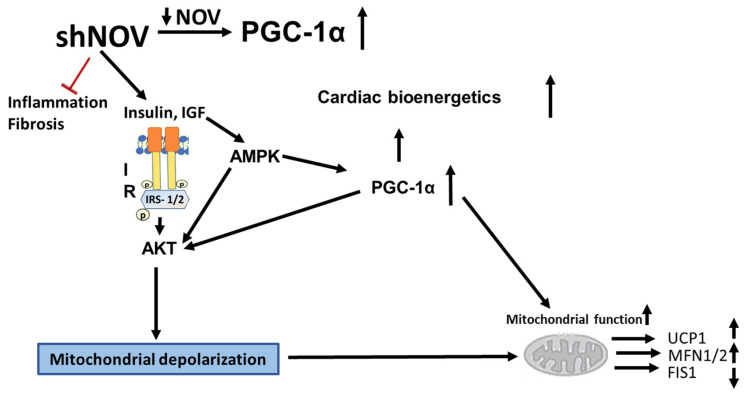
Diagram showing the possible mechanisms by which the silencing of NOV expression improves mitochondrial function, biogenesis, and integrity. The silencing of NOV (shNOV) attenuates HFD-induced inflammation and fibrosis. Insulin sensitivity is improved, leading to the activation of AKT (Protein kinase B). The increase in HO-1 also activates PGC-1α (peroxisome proliferator-activated receptor-gamma coactivator), improving mitochondrial function. The increased AKT activity might lead to increased autophagy. The elevation in autophagy, together with mitochondrial depolarization as a result of oxidative stress, might increase mitophagy. Therefore, damaged mitochondria are removed, and the mitochondrial protein levels of UCP1 (uncoupling protein 1) and MFN1/2 (mitofusion 1 and 2) are increased, while FIS1 (mitochondrial fission 1 protein), expression is attenuated. Ultimately, mitochondrial function is improved.

The inhibition of NOV expression leads to the upregulation of PGC-1α and of key signaling components, resulting in improved glucose tolerance, improved mitochondrial function, and reduced inflammation. At the structural level, these metabolic changes resulted in increased oxygen consumption, reduced adipocyte size, and improved cardiac metabolism and vascular function (Figure 12). The adipocyte-specific deletion of NOV results in an increase in PGC-1α and the activation of mitochondrial proteins. Altogether, these data highlight a key role of the NOV pathway in obesity, mitochondrial function, and inflammation, pointing to the selective targeting of the adipose tissue phenotype as a promising alternative strategy to treat obese patients with cardiovascular co-morbidities. Our study shows, for the first time, that silencing NOV in adipocytes only reduces the cardiac expression of NOV and positively correlates with improved cardiometabolic function.

## Data Availability

The data are contained in the article.
